# Women’s experience of body weight management during and post-pregnancy: a mixed methods approach

**DOI:** 10.1186/s12884-024-07033-6

**Published:** 2024-12-19

**Authors:** Rachel Nolan, Alison M. Gallagher, Alyson J. Hill

**Affiliations:** Nutrition Innovation Centre for Food and Health, School of Biomedical Sciences, Ulster University, Cromore Road, Co. Londonderry Northern Ireland, Coleraine, BT52 1SA UK

**Keywords:** Pregnancy, Post-pregnancy, Weight management, Gestational weight gain, Barrier

## Abstract

**Background:**

Gaining excessive weight during pregnancy has been linked with adverse effects including increased risk of caesarean delivery and postpartum weight retention. Despite these recognised risks, currently no UK/Ireland gestational weight gain (GWG) guidelines exist, women are not routinely weighed throughout pregnancy and consequently, women’s understanding of the importance of weight management during, and post-pregnancy remains poor. The present study explored factors influencing weight change during and post-pregnancy and identified potential opportunities that could help women manage weight during these periods.

**Methods:**

Women aged 18–45 years-old who had an uncomplicated pregnancy within the last 3 years were invited to complete an online questionnaire (*n* = 108) regarding their experience of changes in body weight during and post-pregnancy. Follow on focus groups (*n* = 13 women) were conducted online within the same population to delve deeper into the topic, the sessions were recorded, transcribed verbatim and data subjected to directive content analysis based on the socio-ecological model.

**Results:**

Respondents reported in the online questionnaire, they did not receive enough information around weight management during (81.5%) and post-pregnancy (86.1%). Focus group qualitative data identified barriers to managing weight at each level of the ecological model, at the intrapersonal (e.g., changes in diet), interpersonal (e.g., conflicting advice), environment and society (e.g., COVID-19 pandemic), and institutional and policy (e.g., system failing) levels. Potential strategies to help manage weight were also identified, at the intrapersonal (e.g., need for further information), interpersonal (e.g., support groups), environment and society (e.g., messaging), and institutional and policy (e.g., further follow-up care) levels.

**Conclusions:**

Mothers report receiving insufficient information around weight management, with barriers identified at each level of the ecological model. Highlighting that support and change is needed on multiple levels both during and post-pregnancy, with potential strategies identified, which could potentially inform future studies.

## Background

Gaining excessive weight during pregnancy has been linked with serious adverse effects for both mother and offspring. Including increased risk of, caesarean delivery, large for gestational age baby, gestational diabetes, and development of obesity in mother and offspring [1, 2]. Inadequate gestational weight gain (GWG) has also been shown to have negative outcomes, including an increased incidence of preterm birth and a low-birth-weight baby [3]. Currently within the United Kingdom (UK) there are no specific GWG guidelines, women are not routinely weighed throughout pregnancy [4] and as such, women’s understanding of GWG, and the associated adverse effects remains poor [5]. The United States (US) National Academy of Medicine (NAM) (previously Institute of Medicine, IOM) has published GWG guidelines [6] which utilise pre-pregnancy body mass index (BMI) as the determinant for how much weight a woman should gain during pregnancy. These guidelines were intended for women from the USA and could be applicable for women in developed countries, but may not be representative in a global context, despite them being the most widely referred to guidelines to report GWG rates [6]. A UK based study [7] found that nearly 50% of women gained excessive GWG, these high rates were also reported in a systematic review and meta-analysis [3] of over 1 million pregnant women, using NAM GWG guidelines. Further to these guidelines, in 2023 the World Health Organization (WHO) made a global call for data on GWG, with a view of developing representative global standards for GWG [8].

Pre-pregnancy weight can be a major determinant associated with increased risk of experiencing adverse effects during pregnancy [9]. Women in the overweight or obese categories are advised to lose weight prior to conception [10], and with 55% of pregnancies in the UK being planned [11], this can be anticipated in some cases. With this added risk, most advice around weight management during pregnancy is directed at those in the obese category. Whilst this is necessary, it is important that the message of managing weight during pregnancy is directed to all pregnant women given that excess GWG can result in long and short terms adverse effects despite pre-pregnancy BMI [12]. Further to this, women with the highest BMI (> 40 kg/m^2^) have been found to be less likely to experience excessive GWG when compared to those with BMI 30–39.9 kg/m^2^ [13]. This reaffirms the need for all women to receive advice on weight gain not just those perceived as the highest risk. Those who do gain weight excessively are more likely to retain weight postpartum which in turn results in women entering future pregnancies at a heavier weight, putting them and potential future offspring at further risk of adverse effects [14–16].

Pregnancy is often referred to as a teachable moment [17] as it is a time in a woman’s life when they are more open to health and lifestyle messages to ensure the best outcome for their offspring. However, due to unclear guidelines, lack of resources [18], and potential weight bias from health care professionals (HCP’s) [19], the opportunity to discuss weight management is currently missed [20]. Several interventions aiming to promote healthy GWG implementing either, diet, physical activity or combination intervention have been trialled but overall have not proved successful in reducing risk of adverse effects [2]. Nonetheless, evidence suggests that targeted interventions which are specific to geographic regions and therefore culturally sensitive may be more successful [18, 21]. However, for these to be developed, the barriers to managing weight need to be first identified so that support can be put in place.

When considering the challenges to managing weight and obesity prevention it is important to consider not only at the individual but the overall environment and associated influencing determinants. The ecological model [22] is used widely in this regard to understand the interrelations between individual and social environmental factors by addressing factors on four levels: intrapersonal, interpersonal, environment and society, and institutional and policy. The aim of this study was to explore the factors that influence weight change during and up to three years post-pregnancy, and secondly to identify potential opportunities that could help women to manage weight within these same time frames, which could potentially inform future studies.

## Methods

### Study design

A mixed methods approach was adopted for this study and used both questionnaire and focus group datasets. The questionnaires inform the quantitative data to gain a broad perspective on the topic whilst the qualitative focus groups allow a more in-depth exploration from a sample group of the same population. This study was approved by the Ulster University Biomedical Sciences Ethics Filter Committee (FCBMS-23-002-B), and written informed consent was obtained from all participants.

### Recruitment

Women aged 18–45 years with the ability to understand English, who had a full-term, singleton, uncomplicated pregnancy within the last 3 years were invited to complete an online questionnaire via Research Electronic Data Capture (REDCap) version 14.5.7, regarding their experiences of changes in body weight during and after pregnancy. Women were recruited between April and June 2023, through researcher attending mother and toddler groups within the local area (Co. Londonderry, Northern Ireland). Mother and toddler groups were identified using online sources, the manager of each group was contacted, informed about the study, and asked the women in their group if they were interested. Once groups expressed interest, the researcher attended sessions in person and provided a brief overview of the study and offered women a flyer with a QR code to direct them to the online questionnaire. Recruitment also took place through social media platforms including Facebook, Instagram and Twitter. Furthermore, an email was sent to all staff and students within the university with general information about the study, along with the online link to complete the questionnaire. At the end of the questionnaire participants were invited to participate in a focus group to discuss this topic further.

### Online questionnaire design

The questionnaire was based on a similar survey study [5], who surveyed women during pregnancy on their perspective on weight gain during pregnancy. Results indicated that women did want to receive a target weight gain and further information to be included as part of their antenatal care. The current questionnaire was newly adapted to be suitable for the target audience of women who were up to 3 years postpartum. With the aim of gaining insights into their experiences both during and post-pregnancy and their opinions on current practices around weight management during and after pregnancy. Maternal pre-pregnancy weight and height were self-reported, and BMI was calculated using weight(kg)/height(m^2^). As part of the questionnaire development, the study questionnaire was first piloted on a small group of women (*n* = 3) who met the inclusion/exclusion criteria as well as being reviewed by two registered dietitians for clarity on each individual question.

### Focus groups

A sub-set of participants from the questionnaire study agreed to take part in the focus group sessions, therefore, self-reported data, including BMI was already available. Participants were contacted by the researcher and focus groups were scheduled for a date and time that was most convenient for participants. These semi-structured focus groups were carried out between September and October 2023 by a researcher trained in qualitative research and with previous experience within the area. All focus group sessions were conducted online via Microsoft Teams to ease the burden of participation. The increased use of online platforms was well accepted by all participants as they were familiar and comfortable with the online format [23]. Sessions were between 32 and 90 min (average 59 min) duration depending on number of attendees. A topic guide (see Table 1) with pre-determined questions and topics was used by the researcher for all sessions. Each focus group conversation was split into two parts, first women’s experience of weight management during pregnancy, followed by a discussion on weight management after pregnancy. Overall, focus group sessions were conversational to provide an open and relaxed environment for women to voice their opinions. These sessions were recorded and transcribed verbatim by the researcher. Recruitment continued until saturation of data was reached, meaning that no new ideas or information was offered.

**Table 1:** Semi-structured topic guide for focus groups

Structure	Questions
Introduction	1. Introduce yourself (name, age of your youngest child, how many children you have, and your current occupation e.g. full-time employment, part time, student, maternity leave, full time mother).
During	2. What was your experience with weight during pregnancy?
Pregnancy	3. Were you given any kind of target weight and was this monitored throughout pregnancy?
	4. How did you find managing weight during pregnancy, or was it not something you overly paid attention to?
	5. Did you take steps to control weight and if relevant what were they?
	6. Were you made aware of the risks of adverse events surrounding gaining too much weight during pregnancy, what were some of the issues you were made aware of?
	7. What further support, if any, would have been helpful during pregnancy to help you monitor and maintain a healthy weight gain journey?
Post-	8. Was returning to your pre-pregnancy weight after you had given birth a priority to you?
Pregnancy	9. Were you given any advice or support surrounding weight loss after pregnancy?
	10. Have you found it difficult progressing towards returning to your pre-pregnancy weight and what barriers have you experienced?
	11. What would have been helpful after pregnancy to help you get back to a healthy weight?
	12. When would be the best time to receive that information?

### Data analysis

Questionnaires quantitative data was analysed using Statistical Package for Social Sciences (SPSS) Version 28.0 (IBM Statistics). General characteristics in response to demographic, knowledge and practice questions were analysed by descriptive statistics where categorical data were presented as frequency and percentage and continuous data presented as mean and standard deviation. For the qualitative analysis of focus groups, directive content analysis, as informed by Hsieh and Shannon [24] based on the socio-ecological model was used for data analysis. Whereby one researcher carried out focus groups, transcribed them, then read and re-read to ensure the full breadth of data was understood. Data transcripts were uploaded to NVivo 12 Pro Software (QSR International) for the management and initial coding of data, where data relating to the barriers or potential strategies to managing weight were highlighted. All highlighted data was then mapped to each level of the ecological model, namely: intrapersonal, interpersonal, environment and society, and institutional and policy. Categories and sub-categories within the ecological model were then defined for both during and post-pregnancy. These categories/sub-categories were reviewed and agreed by all members of the research team. Quotes that were most reflective of the categories/sub-categories were selected for inclusion.

## Results

### Questionnaire data

**Fig. 1:**
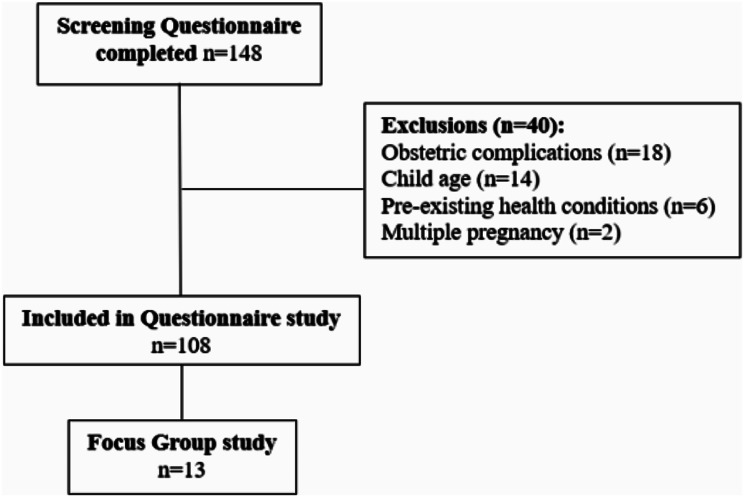
Flow diagram of study recruitment

A total of 148 women completed the initial screening questionnaire between April-June 2023, 40 were excluded, resulting in a total of 108 women included in the study (see Fig. 1).

### Participant characteristics

Characteristics of questionnaire study participants are shown in Table 2. The majority of women were white (91.7%) non-smokers (96.3%), with 37% aged between 31 and 35. Most women (40.7%) were less than one year postpartum when completing the questionnaire, and (53.7%) had two or more children. Over half (56.5%) of respondents held a postgraduate degree (master’s or higher degree qualification). Self-reported pre-pregnancy BMI was available for *n* = 105, (mean 24.3 kg/m^2^, SD 3.6) meaning the majority of women were in the healthy BMI range. Using self-reported pre-pregnancy BMI and reported GWG (*n* = 97), according to the NAM GWG guidelines [6] 25.8% of women gained above and 44.3% were below the guidelines.

**Table 2:** Characteristics of questionnaire participants

Characteristics (*n* = 108)	No. (%) or mean ± SD	
**Age (years)**		
18–25	6 (5.6)	
26–30	21 (19.4)	
31–35	40 (37.0)	
36–40	35 (32.4)	
41–45	6 (5.6)	
**Ethnicity**		
White	99 (91.7)	
**Smoker**	4 (3.7)	
**Pre- pregnancy BMI (kg/m** ^**2**^ )*	24.3 ± 3.6	
GWG (kg)**	10.7 ± 5.3	
NAM Guidelines***		
Below	43 (44.3)	
Within	29 (29.9)	
Above	25 (25.8)	
**Parity**		
1	50 (46.3)	
≥ 2	58 (53.7)	
**Child age (months)**		
0–11	44 (40.7)	
12–23	40 (37.1)	
24–36	24 (22.2)	
**Highest level of education**		
12–14 years	3 (2.8)	
Further education	44 (40.7)	
Postgraduate degree	61 (56.5)	

^*^n = 105 (97.2%) respondents, ^**^n = 97 (89.8%) respondents *BMI* Body mass index, *GWG* gestational weight gain, ***NAM National Academy of Medicine (previously referred to as the Institute of Medicine guidelines^6^), *SD* Standard deviation

### Women’s experiences and opinions regarding weight management during pregnancy

The vast majority (72.2%) of women reported being weighed during pregnancy (Table 3) although 92.6% reported not being given a target weight by a health care professional (HCP). These 92.6% were directed to a follow up question where the majority (48%, see Table 3) responded they would not have liked a target weight. However, 81.5% of participants also reported they did not receive enough information around weight gain overall. Table 4 exhibits women’s opinions on what their ideal idea of care is, with 66.7% agreeing information around weight management should be provided, and the most appropriate time to discuss this would be in the first trimester of pregnancy (57.4%). Further to this, 67.6% of women reported they should be informed on issues surrounding gaining too much weight, correlating with how 52.8% of women could not identify any adverse effects associated with excessive GWG (Table 3). The majority of participants (66.7%) agreed they would like to be informed by a HCP if they were gaining too much or too little weight. A sentiment further supported by 62% of participants acknowledging that discussing GWG should not be avoided due to being considered a sensitive topic.

**Table 3:** During pregnancy- women’s experience of weight management

	No. (%)
**Weighed during pregnancy by a HCP**	
Yes	78 (72.2)
No	24 (22.2)
Not sure	6 (5.6)
**HCP provided a target weight**	
Yes	6 (5.6)
No	100 (92.6)
Not sure	2 (1.9)
**If no**,** would you have liked a target weight**	
Yes	36 (36)
No	48 (48)
Not sure	16 (16
**Received enough information around weight gain**	
Yes	20 (18.5)
No	88 (81.5)
**Aware of the issues surrounding gaining too much weight**	
Yes	51 (47.2)
No	41 (38)
Not sure	16 (14.8)

Sample size *n* = 108, *HCP* Health care professional

**Table 4:** During pregnancy- women’s opinions on ideal care regarding weight management

	No. (%)
**Weight management info should be provided**	
Yes	72 (66.7)
No	17 (15.7)
Not sure	19 (17.6)
**Most appropriate time to be informed about weight gain**	
Before Pregnancy	14 (13)
First trimester	62 (57.4)
Second trimester	11 (10.2)
Third Trimester	2 (1.9)
Every appointment	3 (2.8)
Never	12 (11.1)
Other	4 (3.7)
**Women should be informed on issues surrounding gaining too much weight**	
Yes	73 (67.6)
No	20 (18.5)
Not sure	15 (13.9)
**Should be informed if gaining too much or too little**	
Yes	72 (66.7)
No	36 (33.3)
**Sensitive topic to be avoided**	
Yes	21 (19.4)
No	67 (62)
Not sure	20 (18.5)

Sample size *n* = 108

### Women’s experiences and opinions regarding weight management post-pregnancy

Post-pregnancy (Table 5), 50% of women reported that they had returned to their pre-pregnancy weight. Nearly all (94.4%) women indicated they were not provided with any information on how to return to their pre-pregnancy weight, with 86.1% agreeing there was not enough information around weight loss after pregnancy.

**Table 5:** Post-pregnancy- women’s experience and opinions of ideal idea of care regarding weight management

	No. (%)
**Returned to pre-pregnancy weight**	
Yes	54 (50)
No	53 (49.1)
Not sure	1 (0.9)
**HCP provided info about how to return to your pre-pregnancy weight**	
Yes	6 (5.6)
No	102 (94.4)
**Received enough info around weight loss after pregnancy**	
Yes	15 (13.6)
No	93 (86.1)

Sample size *n* = 108, *HCP* Health care professional

### Focus group data

A total of 47 women provided contact details within the questionnaire to be contacted with further information. Of the 47 women contacted, 19 replied and were still interested in taking part in the study. A final 13 women participated in the focus group sessions between September and October 2023 before reaching saturation of information. Four focus group sessions were carried out, the first contained five participants, and due to last minute dropouts and rescheduling the second consisted of three, and session three and four included two participants. Session five contained only one participant meaning it was a 1-2-1 interview however it followed the same topic guide and structure as all other focus groups. All women who participated were white, non-smokers, further demographics of focus group participants are shown in Table 6.

**Table 6:** Characteristics of focus group participants

Characteristics (*n* = 13)	No. (%)
**Age (years)**	
18–25	1 (7.6)
31–35	6 (46.2)
36–40	6 (46.2)
**Ethnicity**	
White	13 (100)
**Pre-pregnancy BMI category**	
Healthy weight	6 (46.2)
Overweight	7 (53.8)
NAM Guidelines*	
Below	4 (30.7)
Within	6 (46.2)
Above	3 (23.1)
**Parity**	
1	6 (46.2)
≥ 2	7 (53.8)
**Child age (months)**	
0–11	2 (15.4)
12–23	5 (38.5)
24–36	6 (46.1)

BMI Body mass index, *NAM National Academy of Medicine (previously referred to as the Institute of Medicine guidelines^6^)

### Barriers to managing weight during and after pregnancy

The key categories/sub-categories regarding identified barriers to managing weight from the mother’s responses and exemplar quotes are reported in Table 7 and further discussed below. These categories/sub-categories are attributed to all four levels of the ecological model: intrapersonal, interpersonal, environment and society, institutional and policy.

**Table 7:** Mothers’ perceptions on barriers to managing weight during and post-pregnancy including exemplar quotes

	Participant quotations	
	During pregnancy	Post-pregnancy
**Intrapersonal**		
**Changes in diet**		
Craving sugar	*“I just craved sugar just to keep me going” FG2 P#7*	N/A
Pregnancy as an excuse	*“yes*,* I’m pregnant*,* I can eat whatever I want*,* this is great*,* so I kind of used it as an excuse.” FG2 P#6*	N/A
Convenience foods/snacks	*“it was more sandwiches and take away’s and quick foods.” FG5 P#13*	*“snacks would be my downfall umm and again that comes down to needing like an energy and a convenient energy hit during the day and just to deal with the the madness” FG3 P#10*
**Priorities**		
Priority on the baby	*“So I thought*,* right*,* I’m gonna not do the gym anymore because I just didn’t trust myself not to hurt me or the baby*,* to be honest.” FG1 P#2*	*“I suppose it’s just*,* it’s (weight management) not the big priority at that time like*,* and even now*,* whenever like the youngest*,* is over a year*,* the priority is them (kids)” FG3 P#9*
Mental health	N/A	“*So when (second child) came along and I’d had her and I didn’t feel down in the dumps*,* which is so happy to feel nice*,* I didn’t care about the weight I just was so glad to not be low umm and so I just*,* that was always the priority.” FG2 P#8*
**Lack of knowledge**		
Excess GWG is not relevant to them	*“I don’t know if it’s (pause) a reflection of my starting point as well*,* because I’m sure like all the women in this call*,* umm we are we’re all healthy” FG2 P#7*	N/A
Not aware of adverse effects	*“we’ve just chatted there and none of us really knew or ever thought about what possible and adverse effects it could have on the child.” FG2 P#8*	N/A
**Barriers to physical activity**		
Nausea	*“so felt very much nauseated with with (second child) umm to the point that I couldn’t even do exercise” FG2 P#7*	N/A
Postpartum recovery	N/A	“*I found the postpartum recovery quite difficult umm I didn’t feel even pushing a pram was quite difficult for a while. Getting the pram in and out of the car*,* so I was really relying on having somebody else there umm and there’s a bit of anxiety around that as well.” FG1 P#3*
Tired	*“I could probably count on one hand how many times I made it to the gym because I was just so tired. I remember*,* one day I had to have two naps which is a bit excessive umm but yeah” FG1 P#3*	*“come evening time*,* which is the only free time for me*,* I’m just zapped of energy” FG3 P#10*
Financial considerations	N/A	*“I know a lot of private people that do those sort of mummy fit classes*,* and I’m sure you know they make good money from it too*,* but that’s only gonna be open to certain people that can afford it” FG3 P#10*
**Interpersonal**		
**Lack of time**		
Working mother	N/A	*“And then you go back to work and it is just like absolute chaos*,* chaos.” FG4 P#12*
Parity	*“When you’re a new mummy*,* and you don’t have other children at home*,* you can just get up and go to yoga or go to pilates*,* but when you have another child at home*,* you can’t.” FG4 P#11*	*“It (weight management) is very low in the priorities because the kids come first and with the two*,* as we said earlier*,* there is just no time for it” FG3 P#10*
**Conflicting advice**		
Friends and family	*“every week there’s a different food you can no longer have and my mum would have been like*,* that’s fine*,* that’s fine. And then my friends are going no*,* no*,* no.” FG2 P#8*	N/A
Online sources	*“and then you start going down a bit of a rabbit hole with Google*,* how much weight should I gain?” FG1 P#3*	N/A
**Lack of support at appointments**	*“the information’s there you just have to go and get it yourself*,* you’re not told to go and get it you just have to go and find it yourself.” FG1 P#1*	N/A
**Environment and society**		
**COVID-19 pandemic**		
Appointments reduced	*“the 2nd pregnancy as well was during covid. So that kind of influenced that I didn’t have as many appointments I should have so. Yeah*,* umm it was very scary to not have all the appointments” FG5 P#13*	N/A
Amenities closed	*“you know (exercising during covid) that was a worry because I had always been someone who went to the gym and it was closed” FG1 P#4*	*“it was during lockdown as well. So I wasn’t getting out as much there was no classes the way I would have had with the older two*,* and I think that had a massive impact.” FG4 P#11*
**Rural locations**	N/A	*“I definitely don’t*,* I’ve never heard of that (mum and baby fitness classes) like from where I live. But then again*,* we would be like a lot*,* probably smaller community where we are out in the middle of the country.” FG4 P#12*
**Societal Expectations**	N/A	*“your bodies literally been stretched and ripped apart or cut open and everyone then expects you to look like a young 20 year old again*,* like it’s absolutely ridiculous” FG2 P#8*
**Institutional and policy factors**		
**System failing**	*“I know they’re (midwives) really busy and I don’t blame them. I think it’s more the system it’s nothing to do with them*,* it’s the system they’re just doing whatever they have to do or they are told to do.” FG5 P#13*	*“there’s no follow up really after*,* if you had a knee operation you would get physio if you had a sprained ankle you’d get physio you really have to beg for it when you have a baby.” FG1 P#3*

*FG* Focus group, *GWG* gestational weight gain, *N/A* not applicable, *P* Participant

### Intrapersonal

#### Changes in diet

Multiple changes to the diet were identified during pregnancy. This included craving sugar, where women felt cravings were out of their control, women also referred to needing sugar to obtain a quick source of energy or to combat nausea. Nonetheless, many women did acknowledge being aware they were not eating for two, and recommended calories do not significantly increase during pregnancy. However, they did see pregnancy as an excuse to relax their eating habits, and a time they could eat whatever they wanted. An increase in convenient foods, meaning take- away foods, energy bars and snacks, was observed both during and after pregnancy, this was linked with other categories including being tired, lack of time and craving sugar. These factors were reported as reasons to need something quick and easy which the participants recognized were more liable to be an unhealthy option.

#### Priorities

Weight management was not considered a main priority by most participants during pregnancy. In contrast ensuring the baby was healthy was their main focus. This priority on baby continued post-pregnancy with women continuing to put priority on the baby and ensuring they were cared for before themselves. However, women did consider themselves when discussing mental health post-pregnancy and the need to have their mental health in a good place before they could prioritise anything else. This was in relation to previous experiences of mental health being negatively affected and acknowledging the importance of preventing that.

#### Lack of knowledge

Women were not made aware of the adverse effects associated with excessive GWG from any HCP, this resulted in a lack of knowledge during pregnancy regarding the adverse effects. This included any adverse effects excess GWG can have on the offspring, which was of particular concern to the women, therefore they were surprised that they were not made aware of this. Furthermore, they did not think excessive GWG was relevant to them because they did not have obesity when entering pregnancy and therefore did not consider weight as an important factor.

#### Barriers to physical activity

Women presented multiple barriers which prevented them from taking part in physical activity during pregnancy. This included feeling nauseous, as women did not feel capable of exercising due to the nausea. Some women did also note nausea influencing their eating habits from avoiding food to opting for plain and salty foods they could consume. Being tired was experienced both during and post-pregnancy, where during pregnancy it was identified as a symptom of pregnancy. Postpartum women noted sleep deprivation due to caring for a newborn baby and caring for other children, meaning women were not getting adequate sleep meaning they did not have the energy to exercise. Not being able to exercise confidently was linked with both postpartum recovery and financial considerations. Women who gave birth via caesarean section experienced pain at their scar site, and those who reported suffering with diastasis recti had a lack of core strength. This resulted in lack of confidence as to what was safe, therefore inhibiting them from engaging in physical exercise. Pelvic floor being weak was also related to postpartum recovery and lack of confidence where exercise could result in a *“*leak*”* causing embarrassment and discouraging them from exercising. This was further enhanced by financial considerations due to many exercise and physio classes being an added cost that not everyone would be able to afford and therefore have no advice on how to exercise safely.

### Interpersonal

#### Lack of time

One of the main divides within the cohort was parity, for 6 women this was their first child and the remaining 7 had two or more children. Those with two or more children heavily emphasised the lack of time they had to be concerned with weight management due to caring for two or more children. They also noted how it was easier to lose weight gained after their first child but having additional children made it difficult with conflicting schedules for each child. Those with only one child did not resonate with having a lack of time and were overall more accepting to welcoming advice around weight management. Again, mostly women with two or more children identified work commitments as a barrier contributing to lack of time to focus on weight management.

#### Conflicting advice

During pregnancy, mothers identified conflicting advice from varying sources including friends and family and online sources which included Google and Facebook groups. Friends and family showed generational differences as the participants own mothers use advice they were given when they were pregnant whilst friends offer differing updated advice. This misinformation was further driven by Google offering many different sources and therefore conflicting advice. However, women did acknowledge positives through both of these sub-categories, with how family and friends can also provide reassurance, and the value of using the NHS website as a reliable source where it was stated: *“if you’re googling anything*,* google it and then put NHS after and that filters out all the other stuff.” FG2 P#7*.

#### Lack of support at appointments

During pregnancy, women reported a lack of support at appointments with regards to weight management. Women commented on not being provided support surrounding weight management and even feeling like the topic was dismissed with woman stating, *“I always had that worry that I had gained too much*,* but then to just have it completely dismissed sometimes find like a little bit of*,* um*,* (small pause) I can’t explain it*,* but do you know something’s completely dismissed you don’t feel like you’ve been heard” FG1 P#3*. This could be through either not being provided advice on it, or women feeling like the advice was there, but you had to find it yourself (see Table 7 for quote).

### Environment and Society

#### COVID-19 pandemic

Due to the timing of this study, many women were affected by the COVID-19 pandemic, which meant that the number of scheduled pregnancy appointments were reduced or kept brief to not put women at risk of contracting corona virus. Women identified this as a possible reason why weight was not discussed, especially amongst first time mothers as they had no experience to compare it to. Further to that, it was identified both during and post-pregnancy that amenities were closed. As a result, women could not carry out their usual habits including, going to the gym, fitness classes or mother and toddler groups. These are activities involving exercise that women would have usually walked to, therefore affecting physical activity habits. This again especially resonated with mothers who had other children, whereas first time mothers did not have previous experience to compare the difference with.

#### Rural locations

Women expressed limitations on accessing amenities such as exercise or physio classes depending on where they were living, as those in rural communities may need to commute a considerable distance to access these. This barrier was highlighted in relation to post-pregnancy as with a small baby it can be difficult to travel long distances on their own. It was also acknowledged that in the darker evenings rural locations do not have safe roads to walk due to having no streetlights, therefore inhibiting safe physical exercise.

#### Societal expectations

Women perceived the expectations society put on them as an unrealistic and added pressure and therefore a barrier to managing weight. Mothers reported that after pregnancy they were expected to look the way they did pre-pregnancy, however many women noted frustration at this as it was considered an unrealistic expectation. Furthermore, if they did not lose excess weight quickly, they became frustrated in that their methods were not effective. Women also noted their bodies are now different, with some women detailing they are close to their pre-pregnancy weight, but their body shape has changed meaning they will never look like they did pre-pregnancy.

### Institutional and Policy factors

#### System failing

Women identified an overarching theme of the system failing both during and post-pregnancy, where they identified there was something missing in terms of a lack of physical and mental wellbeing support. During pregnancy women did not place blame on the midwives as it was not their area of expertise. Therefore, they acknowledged something else was missing that should be in place to support women with regards to weight management. The majority of the participants placed heavy emphasis on the system failing post-pregnancy. Women discussed the lack of support and follow up care post-pregnancy along with being left to their own devices and mothers feeling forgotten by the system. With one woman stating: *“we are completely forgotten about whether it was the section*,* whether it was stitches*,* whether it was natural like we are*,* there is no*,* the postnatal care is diabolical to say the least.” FG2 P#8*.

### Strategies to help manage weight during and after pregnancy

When asked what would have been helpful during and after pregnancy to assist and support in managing weight, women did acknowledge how there is no one-size fits all solution. This made it difficult for the women to answer the question: *“it’s gonna be different for everybody and different depending on*,* you know*,* which pregnancy it is as well*,* so it’s hard to say what would work.” FG3 P#10*. Opinions were also divided depending on if this was the participants first child, as first-time mothers were more open to support. Whereas mothers with multiple children raised more barriers with regards to time available, and even acknowledged how their attitude would have been different in their first pregnancy relating to the current first-time mothers. There was also further openness overall to receiving support with weight management post-pregnancy. Meaning emphasis was heavily put on the post-pregnancy period and conversations were often redirected to post-pregnancy by the participants.

The key categories/sub-categories regarding mothers’ opinions on what would be helpful to manage weight and exemplar quotes are reported in Table 8 and further discussed below. These again are mapped to each level of the ecological model namely: intrapersonal, interpersonal, environment and society and institutional and policy level.

**Table 8:** Mothers’ views on strategies to support weight management during and post-pregnancy including exemplar quotes

	Participant quotations	
	During pregnancy	Post-pregnancy
**Intrapersonal**		
**Need for further information**		
Components of GWG	“They explain all the baby puts on 5 pounds from this date. So, but they don’t explain how it affects Mom. So maybe an explanation” FG4 P#11	N/A
GWG guidelines	“they should record your weight and they will tell you how much weight you are expecting to gain during the pregnancy and what is going to happen with your (pause) well, the first trimester will not put so much weight, the second one you might, but this is the limit.” FG5 P#13	N/A
Signposting	“go to this website to get a bit more information or whatever, just information and then sign posting in a simple way” FG1 P#5	“maybe there could be like a leaflet, you know guidance or something that like here’s the basics if you want more information then you can go and look online and that might sort of hit both sets of people from that.” FG3 P#10
Practical cooking methods and recipes	N/A	“So these are some easy, quick meals that you could make with a new born, takes 5 minutes with a newborn, you know” FG2 P#8
**Interpersonal**		
**Support groups**	N/A	“there is definitely a lot of value to anywhere where there’s other mums umm so I don’t know how that could have a bit of a weight management focus, but just getting other people’s experience is really helpful.” FG1 P#3
**Environment and society**		
**Messaging**		
Realistic expectations	N/A	“yeah, realistic expectations you’re gonna be so sleep deprived, you know. Yeah, you’re gonna survive on whatever you can get.” FG1 P#4
Positive healthy Mum messaging	N/A	“ having that strength to to be a mum, I think is probably nice messaging to to send out, not necessarily around your pre pregnancy weight but yeah, being strong enough for what life is gonna throw at you now.” FG2 P#7
**Institutional and Policy factors**		
**Regular weighing**	“I think umm regular weighing would definitely and tell me if I’m keeping on good track. Yeah, that would have been helpful…. obviously not at every appointment, but every now and then more regular, more regular than the previous pregnancy, because that was just at the start and at the very end, and it was just such a shock, uh, seeing that massive weight gain” FG1 P#1	N/A
**Further post-pregnancy follow ups**	N/A	“You should have a three month and then a six month to check that you’re progressing through it and and that you’re OK. It it should be less, a little less about the like, a child needs a happy mum, a healthy mum and we are completely forgotten about” FG2 P#8

*FG* Focus group, *GWG* gestational weight gain, *N/A* not applicable, *P* Participant

### Intrapersonal

#### Need for further information

When discussing GWG during pregnancy, women recognised gaps within their knowledge on the topic and a need for further information. One area identified as requiring further information was the components of GWG. Women knew how much their baby was growing but weren’t aware of how other components such as extravascular fluid and blood volume were also contributing to GWG. It was therefore reported an explanation would have been helpful, with some women wanting to know how much weight they should be gaining during pregnancy. This was linked with signposting, where during pregnancy women noted if they did think they were gaining excess weight they would like signposting to helpful resources or advice. Signposting was also emphasized post-pregnancy as previously mentioned women acknowledged how every weight management journey is different and different women want to focus on different aspects. Therefore, signposting meant women could identify which factor they needed more information or support with and know where the best place is to go for that advice making it a more individualized approach. Signposting specific to their local area was also reported so they were aware of all classes and support available to them. One of the aspects to possibly be directed to was practical cooking methods and recipes. This involved suggestions such as using the slow cooker so meals are ready when family come home from work, as well as quick easy recipes that would be suitable for the whole family.

### Interpersonal

#### Support groups

Relating to other women going through the same situation and sharing experiences was valued by women post-pregnancy. Whilst women did acknowledge attending mother and baby groups, they noted how these were focused on baby, there wasn’t groups focused on supporting mothers. Women suggested these could be in person, online or through social media but to be focused on mothers so that topics such as weight management could be discussed as another form of support for mothers. Exercise classes that you can bring your baby to whilst exercising with other mothers were also discussed to be made available.

### Environment and Society

#### Messaging

When considering what would have been helpful in managing weight post-pregnancy, women highlighted the importance of promoting positive messaging. This included realistic messaging which acknowledged barriers to managing weight. For example, acknowledging the new demands on life impacting the ability to lose weight immediately, such as lack of energy to exercise due to sleep deprivation. Although the main focus of this present study was weight, many women felt messaging which focused solely on weight would not be sufficient to encourage women to engage. Rather messaging around “being strong and healthy to be ready for life with a baby” would be more likely to engage these women. Such positive messaging would serve to encourage women to put some focus back on their own health and wellbeing and would also remove any perceived guilt around looking after themself rather than being solely focused on their baby.

### Institutional and Policy factors

#### Regular weighing

Many women did think regular weighing would have been beneficial to them in order to see a steady progression throughout pregnancy and be able to make changes in a timely manner, if necessary, due to being aware of their weight. However, some women also raised concerns, these included the approach of the HCP weighing them, that they would place emphasis around weight making them feel guilty. Therefore adding another thing to worry about along with all the other worries during pregnancy, with one woman discussing regular weight stating: *“it would have given me more worries than I needed to have and I don’t know how you land that in a really tactful way.” FG1 P#5*.

#### Further post-pregnancy follow ups

Women noted the only follow up appointment post-pregnancy being at 6 weeks postpartum, which they deemed not a suitable time to discuss weight. Therefore women felt there should be further follow up appointments to check in with mothers’ wellbeing and as part of that their weight management. Many women identified going back to work as a time they would have been more accepting to advice and support as their routine was changing anyway. This differed depending on women’s situation, so women also identified around six months post-pregnancy to be a more appropriate time. Women could not identify exactly what this would look like but something that checks in with mothers at regular intervals post-pregnancy to ensure they are progressing positively.

## Discussion

In this mixed methods study women’s experience during and post-pregnancy regarding weight management was explored. The initial quantitative side of this study provided an initial insight into women’s experiences with weight management and their opinions on what their ideal idea of care would be. Qualitative data delved deeper into personal experiences of women within the normal and overweight BMI categories. Several barriers to managing weight were identified, as well as women’s opinions on what would be useful in the future to help support mothers during and after pregnancy. These were mapped to one of the four levels of the ecological model.

Questionnaires identified that most women were not provided with a target weight during pregnancy, which is to be expected as there are currently no specific GWG guidelines for use within the UK/Ireland [10]. It is important to note, other countries do provide target weights such as America utilising the NAM [6] guidelines, and countries within Latin America such as Chile and Brazil using Atalah’s curve [25, 26]. However, whilst receiving a target for GWG did not receive a positive reaction from participants within this study; the majority of women did report not receiving enough information around weight management during pregnancy. Further findings identified that most women would welcome information around weight management during pregnancy and reported they would like to be told if they were gaining too much or too little weight. This idea was further reinforced through focus groups opinions, where many women did suggest regular weighing throughout pregnancy would have been beneficial, if it was part of routine care and would have reassured them they were progressing positively. Routine weighing had originally been part of clinical practice but was phased out due to reports that it was no longer useful and caused anxiety to pregnant women [27]. More recent findings indicate women no longer hold this view and would be accepting to regular weighing [4, 28, 29], but this can be dependent on there being a purpose to the weighing [30]. However, it is important to note that not all women held this view and felt it was another factor to consider on top of everything else. They also placed focus on the importance of how the HCP carrying out the weighing approached the topic, therefore extra training for HCP could be advised. Questionnaire participants identified the first trimester as the best time to initially receive information around weight management. This correlates with advice from literature [18] as the first trimester being the best time to capitalize on the opportunity for a teachable moment and have the biggest impact. The lack of information provided to women during pregnancy was evident further, where within the questionnaire the majority of women were not aware of and could not identify any adverse effects associated with excess GWG.

Lack of knowledge was also identified through focus group results of this study where it was identified that women perceived excess GWG was not relevant to them, however excess GWG can result in adverse effects for anyone regardless of BMI category [12]. In addition, whilst ensuring the baby was healthy was considered a main focus, women did not identify the correlation between weight management and positive outcomes for their baby’s health. This lack of priority on weight has been reported elsewhere [30], with weight gain being seen as an inevitable part of pregnancy and therefore not a priority. Further to this, over half of the women within this cohort were in the overweight BMI category, meaning weight should have been something to consider for them. However, it has been reported [31] that midwives would not be concerned with a high BMI due to how common it is in society, meaning they are less likely to discuss the topic. This could be linked with the lack of support women reported throughout pregnancy at their appointments with regards to weight management. In addition, they noted weight related advice was not personalised to them and when weight was brought up by participants, HCPs’ were not concerned by it [32]. Instead, they were directed to general online resources feeling like they had to obtain the information themselves. Participants did report on receiving conflicting advice on GWG and nutritional advice due to obtaining information from commonly used sources including online sources [20] as well as family and friends [33]. Health information from online sources is generally inconsistent and can influence women’s behavior in a negative way which potentially result in worse health outcomes [34]. Participants reported finding conflicting sources on the internet as well as differing opinions from family and friends to be confusing and frustrating when trying to find reliable and consistent information.

Participants in the present study also reported common universal barriers to managing weight around pregnancy including cravings, nausea and tiredness [35]. Cravings are commonly experienced during pregnancy and have been recognized as a main driver to increasing energy intake during pregnancy with sweet foods and beverages being main contributors [36, 37]. Women also reference using “sugary foods” as a “quick” source of energy to combat tiredness and general lack of energy. Tiredness was also recognized as a sub-category within barriers to physical activity where being tired is a common symptom of pregnancy [38] and adjusting to life with a baby after pregnancy is also recognized to increase tiredness [39]. Tiredness and other identified factors such as lack of time resulted in women relying on convenience foods and quick easy unhealthy snacks both during and post-pregnancy, as women noted not always having enough time to make homemade healthy options, especially for themselves. Pregnancy was also seen as an opportunity to relax habitual dietary habits. A common misconception during pregnancy is that you are “eating for two” and whilst women identified this as false through their own knowledge or being informed by a HCP. They did still use pregnancy as a time to relax stricter dietary habits which has been commonly reported in other qualitative studies [40].

An observation made from the focus group work was that the views of participants differed depending on if they were primigravida or multigravida. Similar research [41] also acknowledged, women with two or more children referred to lack of time to consider weight management due to caring for other children as a vast barrier. This was evident also through women’s willingness to accept further support both during and post-pregnancy where those with only one child were more open to support whilst those with two or more children presented more barriers. These barriers included several points around having two or more children as well as being a working mother and the demands associated with that resulting in lack of time. This has been identified previously in the demand around work life balance and resultant lack of time for exercise [42, 43]. Another observation regarding multigravida mothers was experiences during covid, as those with two of more children had experiences both within and before covid meaning they could make direct comparisons whereas those in their first pregnancy could only speculate. Women did identify the reduced access to health care throughout the pandemic [44], and with that the increased uncertainty around their pregnancy [45], as they were not receiving the same reassurance provided with normal routine appointments, meaning less time for discussing weight management. However, it is important to note that due to COVID restrictions and amenities being closed, many women actively decided to go for a walk every day for exercise, displaying they understood the importance of exercising during pregnancy.

Postpartum recovery was an identified barrier to physical activity as women identified a prolonged period of time after birth that they physically could not exercise due to general recovery including, caesarean section scars, diastasis recti and weak pelvic floor. Following on from this, financial considerations were also noted, as postpartum physios and exercise classes advertised to mothers were not affordable for everyone [46]. Meaning some women were unsure about when it would be safe to return to exercise and at what intensity to not injure themselves further. This calls attention to the need for recommendations for returning to exercise postpartum safely [47]. Postpartum classes that did exist within the local area, women reported not being aware of these and suggested signposting within the local area about available classes to encourage participation. Women did comment that current mother and baby classes were focused on baby with no priority on mum. However, participants did see the value in these groups through receiving reassurance and support from women in the same situation. It was suggested that support groups focused on mothers that could also have a weight management and general health focus on women could be beneficial for weight management. Rural locations were also related to this, due to having to travel long distances to avail of classes, which was not deemed viable with having small children to care for.

Post-pregnancy, questionnaire participants reported not being provided any information from a HCP about how to return to their pre-pregnancy weight and overall they did not receive enough information around weight management post pregnancy. Lack of follow-up care post-pregnancy was a huge consideration for focus group participants, this has been highlighted previously [48, 49] and now further evidenced in this study. Focus group participants reported only one routine follow up at six weeks post-pregnancy which many criticized as not being thorough enough in terms of physical and mental wellbeing. Six weeks postpartum was also acknowledged as not an appropriate time to discuss weight management. Participants highlighted how six months postpartum or when returning to work were periods, they would potentially have been more open to advice on weight management. This could be a focal consideration for many women as an opportunity to understand the importance of entering pregnancy at a healthy weight within the inter-pregnancy window [50]. As pre-pregnancy weight has been reported to have the greatest effect on most clinical outcomes [9, 51], making this an opportune time for women to make positive changes to their weight. Participants within this study also identified how this was not to put blame on HCPs, but instead the system should have procedures in place to follow up with women and provide advice and support. One factor women in this study did emphasise as helping them lose weight post-pregnancy was, breastfeeding. Whilst some studies do disagree with this [52], it is widely reported that breastfeeding can aid in weight loss postpartum and should be encouraged [53, 54], as reported in this study.

### Strengths and limitations

This was a small study, and whilst in person recruitment from mother and toddler groups were recruited only from local areas, social media and the university email allowed for recruitment to reach a much wider audience. Limitations observed in this study include the lack of diversity within the cohort, as most women were of white ethnicity meaning the sample was not representative of global population. However, the most recent Census report in Northern Ireland [55], where recruitment was carried out, reported nearly 97% of the population was white therefore meaning recruitment was representative of the population. Further research would need to be conducted to include more diverse population views. Within the questionnaire cohort the majority of women had a BMI within the healthy range. This could be a potential limitation since these women may not have experience of issues related to weight and therefore have limited knowledge of the area. However as outlined in this manuscript excess GWG can lead to adverse effects, despite pre-pregnancy BMI and therefore does need to be considered by all women. Furthermore, whilst participants self-reported their height and weight for calculation of BMI and GWG, the accuracy of such self-reported pre-pregnancy BMI is known to be highly accurate for research purposes [56]. However, those in overweight and obese BMI categories have been found to be less reliable when self-reporting weight [57]. Furthermore, when considering reported GWG using the NAM guidelines this study found that the majority of women gained below the recommended guidelines in contrast to UK based data [7]. Therefore, it is important to recognise how underreporting of body weight (and subsequent BMI calculations) then result in a lack of accurate data for reported NAM guidelines as they are directly linked. Level of education was also extremely high within this cohort, with 97.2% of participants having completed a technical college, undergraduate degree or higher. This reinforces the lack of knowledge around weight management during and post-pregnancy even in a highly educated cohort of women. Nevertheless, it does also mean that these findings cannot be fully extrapolated to all women of childbearing age.

## Conclusion

In summary, this study shows that women are keen to receive information on weight management during pregnancy, however the term ‘target weight’ does not receive a positive reaction and suggests that any information provided to women during pregnancy should perhaps be more health based rather than discussing weight and using terms focused on weight. Participants placed emphasis on the lack of follow up support and information on weight management post-pregnancy highlighting a need for more information from reliable sources such as HCPs to help women manage weight after pregnancy. Barriers to managing weight were identified at all four levels of the ecological model indicating that support and change is needed on multiple levels both during and post-pregnancy. Finally, opinions on what support and change could look like were also offered which could potentially inform future studies. This work adds to the body of evidence which supports the provision of additional support to women during and post-pregnancy and again highlights the need for a local approach when considering the barriers and enablers of managing weight.

## Data Availability

The datasets generated and/or analysed during the current study are not publicly available due to the ethical concerns related to the identifying personal information but are available from the corresponding author on reasonable request.

## Abbreviations

BMI: Body mass index
FG: Focus group
GWG: Gestational weight gain
HCP: Health care professional
IOM: Institute of Medicine
NAM: National Academy of Medicine
N/A: Not applicable
P: Participant
REDCap: Research Electronic Data Capture
SD: Standard deviation
SPSS: Statistical Package for Social Sciences
UK: United Kingdom
US: United States
WHO: World Health Organization
